# Analysis of cells of epithelial, connective tissue and immune differentiation in HPV-positive-, HPV-negative oropharyngeal carcinoma and normal oropharyngeal tissue by immunofluorescence multiplex image cytometry: a preliminary report

**DOI:** 10.1186/s12885-023-11440-x

**Published:** 2023-11-27

**Authors:** Aris I. Giotakis, Annette Runge, József Dudas, Rudolf Glueckert, Timo Gottfried, Volker H. Schartinger, Johanna Klarer, Avneet Randhawa, Eleonora Caimmi, Herbert Riechelmann

**Affiliations:** 1 Department of Otorhinolaryngology – Head & Neck Surgery, Medical University of Innsbruck, Anichstrasse 35, Innsbruck, 6020 Austria; 2 University Clinics Innsbruck, Tirol Kliniken, Anichstrasse 35, Innsbruck, 6020 Austria; 3 Department of Otolaryngology, Rutgers University, New Jersey Medical School, Newark, NJ USA

**Keywords:** Image cytometry, Fluorescent antibody technique, Epithelial cells, Connective tissue, Leukocytes, Oropharyngeal neoplasms, Human papilloma virus

## Abstract

**Background:**

Epithelial, connective tissue and immune cells contribute in various ways to the pathophysiology of HPV positive (HPV+) and HPV negative (HPV-) oropharyngeal squamous cell carcinoma (OPSCC). We aimed to investigate the abundance of these cell lineages and their coexpression patterns in patients with HPV + and HPV- OPSCC.

**Methods:**

We used a 4-channel immunofluorescence-microscopy technique for the simultaneous detection of three direct-conjugated antibodies (pancytokeratin, vimentin and CD45/CD18) and DAPI (4’,6-Diamidin-2-phenylindole) in formalin fixed paraffin-embedded tissue samples (FFPE) of patients with HPV + and HPV- OPSCC, and of control patients. Image acquisition and analysis were performed with TissueFAXS and StrataQuest (TissueGnostics, Vienna, Austria), respectively, in tumor cell clusters/stroma in OPSCC specimens and epithelial layer/lamina propria in control specimens. Cell populations were created based on antibodies’ coexpression patterns. Isotype and positive controls were examined for plausibility.

**Results:**

The proportion of cells of epithelial differentiation in tumor cell clusters was higher in HPV + OPSCC (55%) than in HPV- OPSCC samples (44%). The proportion of connective tissue cells in tumor cell cluster was lower in HPV + OPSCC patients (18%) than in HPV- OPSCC patients (26%). The proportion of immune cells in tumor cell clusters was higher in HPV + OPSCC patients (25%) than in HPV- OPSCC patients (18%). The percentage of anaplastic, potentially de-differentiated cells, was 2% in control patients, and it was higher in HPV- OPSCC (21%) than in HPV + OPSCC samples (6%).

**Conclusions:**

This study provided the first quantitative data for the abundance of cells of epithelial, connective tissue and immune differentiation, in patients with OPSCC and control patients. The abundance of these different crucial cell populations was consistently originating from the same tissue sample. De-differentiation of tumor cells was higher in HPV- OPSCC than in HPV + OPSCC. In tumor cells clusters, the antitumoral host immune response was higher in HPV + OPSCC than in HPV- OPSCC, whereas the fibroblast response was higher in HPV- OPSCC than in HPV + OPSCC. This study contributed to the understanding of histopathologic differences between HPV + OPSCC and HPV- OPSCC patients.

**Supplementary Information:**

The online version contains supplementary material available at 10.1186/s12885-023-11440-x.

## Background

There are two subtypes of oropharyngeal squamous cell carcinomas (OPSCC): human papilloma virus (HPV) positive (+) and negative (-) [1, 2]. Patients with HPV + OPSCC have significantly better prognoses than patients with HPV- OPSCC [3–6]. Accumulating data indicate that the tumor microenvironment plays an important role in the pathogenesis and development of treatment resistance. The cellular composition and the tumor/stroma ratio both play critical roles in invasive growth patterns and drug resistance [7].

Stromal fibroblasts, especially the so-called carcinoma-associated fibroblasts subpopulation, were described as drivers of invasive cancer growth [8]. Recently, Bolt and coauthors conducted a study using 2D and 3D modeling of interaction of OPSCC with fibroblasts. They reported that only HPV- OPSCC cells induce a fibroblast response that supports cancer migration and invasion in vitro [9]. Moreover, immune cells infiltrating the tumor microenvironment may represent an antitumoral host immune response against antigens expressed on epithelial cells [10, 11] and influence the clinical outcome [12]. Furthermore, cytokeratin, as expressed on the surface of epithelial cells, plays an important role in cell stabilization and intracellular signaling [13, 14]. Several studies report a significant association between cytokeratin 7 expression and OPSCC HPV tumor status [15–17].

Data on the proportions and differentiation of epithelial cells, connective tissue cells, and leukocytes in OPSCC are scarce. Recently, immunofluorescence multiplex image cytometry has been used to describe proportions of these cell populations in upper airway mucosa. [18] This technique allows for the quantification of various cellular subtypes and the recognition of patterns of co-expression in formalin-fixed paraffin embedded (FFPE) tissue samples [19–21]. It also overcomes the limitations of established methods of cellular quantification, i.e., immunohistochemistry and flow cytometry, by eliminating the need for individual investigation of intact solid tissue slides.

Here, we aimed to investigate the relative abundance of these cell populations in patients with HPV + OPSCC and HPV- OPSCC by immunofluorescence multiplex image cytometry. As control tissue, we used normal oropharyngeal mucosa of patients with sleep related breathing disorders. For the detection of cells of epithelial, connective tissue, and immune differentiation, we used the epithelial marker pancytokeratin, the connective tissue marker vimentin and the leukocyte markers CD45/CD18, respectively. Tumor cell clusters and stroma were manually outlined and separately examined in patients with OPSCC. Epithelial layer and lamina propria (LP) were similarly assessed in control patients.

## Methods

### Study population

The study was conducted with standards of Declaration of Helsinki and approved by the local ethics committee (UN3678). Informed consent was obtained from all subjects prior to sample collection. For this study, we intended to analyze a convenience sample of eight patients with HPV + OPSCC, eight patients with HPV- OPSCC, and eight control patients. This convenience sample consisted of FFPE samples drawn from the tissue biobank of the Department of Otorhinolaryngology – Head & Neck Surgery, Medical University of Innsbruck, Austria.

### Patients with OPSCC

The FFPE samples were produced from tumor biopsies taken during panendoscopy for tumor staging. Inclusion criteria were histologically confirmed squamous cell carcinoma of the oropharynx, known high risk HPV-status by p16 immunohistochemistry [22], and patient consent to use anonymized data and tissue samples for scientific investigations and publications. A commercial in vitro diagnostic certified assay containing a ready-to-use prediluted mouse monoclonal antibody was used for p16 detection (CINtec® Histology V-Kit, Roche Ventana, Tucson, AZ, USA) [23]. The definition of an HPV + tumor was based on the overexpression of p16. The cutoff point for p16 overexpression was diffuse (> 75%) tumor expression, with at least moderate (+ 2/3) staining intensity [24].

### Control patients

FFPE samples of patients with sleep related breathing disorders without head & neck cancer were drawn from the biobank. The patients had undergone uvulopalatopharyngoplasty, in which a strip of healthy human oropharyngeal mucosa was resected. This tissue is usually discarded. In this investigation, it was used as control tissue. HPV-status was not tested in control patients.

### Positive and isotype controls

Positive- and isotype controls served to check the results of immunofluorescence multiplex image cytometry for plausibility. Positive controls for cytokeratin included the tumor cell line CAL-27 (DSMZ number: ACC 446; DSMZ, Leibniz Institute, Braunschweig, Germany) [25] and the isolated epithelial layer of control samples of two patients. Four million cells of tumor cell line CAL-27, cultivated in DMEM Medium, were collected by centrifugation at 290 g (10 min, at 4 ° C), fixed in 4% formaldehyde solution overnight, centrifuged at 400 g for 10 min, at 4 ° C, rinsed in PBS, and embedded in paraffin. Five µm sections were also made and immunofluorescence staining was performed [25, 26]. A human gingival fibroblast (hGF) cell line (CLS order number: 300,703; CLS Cell Lines Service, Eppelheim, Germany), cultivated in DMEM/F12 medium, served as positive control for vimentin [27]. A human B-cell lymphoma (non-Hodgkin lymphoma) sample and a human tonsil (sample of possibly inflamed tonsil obtained from the operating theater through tonsillectomy for research purposes) served as positive controls for CD45/CD18 expression. Positive controls were stained with all three antibodies and 4’,6-Diamidin-2-phenylindole (DAPI). Positive controls were expected to yield high intensities for the targeted antigen and low intensities for the others.

Antibodies of the same isotype, clonality, conjugate, and host species as the antibodies used to detect cytokeratin, vimentin, and CD45/CD18, which targeted molecules that were not present in the sample, were used for isotype controls. Commercially available isotype antibodies for the three fluorochromes were used. The characteristics of the isotype antibodies are listed in the first table of the study of Giotakis and coauthors [18].

### Specimen embedding and cutting

Specimen embedding and cutting were described in detail in the study of Giotakis and coauthors [18]. Briefly, the samples were transferred to Modified Eagle’s Medium with Earle’s Salts without L-glutamine (PAA Laboratories GmbH, Pasching, Germany). Biopsies were sectioned to 5-µm thickness using an HM 355 S microtome (Microm, Walldorf, Germany).

### Antigen retrieval and immunostaining

Co-labeling of the epithelial marker pancytokeratin, the fibroblast marker vimentin and the leukocyte marker CD45/CD18 was achieved using direct-conjugated primary antibodies and a fully automated immunostaining system (Ventana Discovery classic, Roche, Mannheim, Germany). The antibodies pancytokeratin, vimentin, CD45 and CD18 were directly coupled to the fluorescent dyes AlexaFluor 488 (AF488), eFluor 570, AlexaFluor594 (AF594; Biolegend, San Diego, USA) and AF594 (Bioss Antibodies, Woburn, USA), respectively. As a nuclear counterstain, we used DAPI (1:46.000, Thermo Fisher Scientific, Darmstadt, Germany) [28]. Antibody details are listed in in the first table of the study of Giotakis and coauthors [18]. Immunostaining, autofluorescence reduction and test for channel spillover were described in detail by Giotakis and coauthors [18].

### Image acquisition

For image acquisition, the TissueFAXS PLUS system (TissueGnostics, Vienna, Austria) was used [18]. The fluorescence microscope was equipped with four bandwidth filters to detect fluorescence of different wavelengths in four channels, which corresponded to fluorophores DAPI, AF488, eFluor570, and AF594. The characteristics of the microscope filters and fluorophores are listed in the second table of the study of Giotakis and coauthors [18]. The fluorescence intensity in the four channels was imaged sequentially in 16-bit monochromally and could be merged into one image. False-colors were arbitrarily chosen for each channel: green was used for cytokeratin (AF488), red for vimentin (eFluor570), and yellow for CD45/CD18 (AF594); blue was reserved for DAPI (DAPI). After a preview of whole slides with a 2.5x lens, the software function ‘automatic tissue detection’ was applied. The detection run was done on the entire preview image, which was acquired in full size using a 40x air objective [18].

### Image analysis

We used the image analysis software StrataQuest (TissueGnostics) for image analysis. The method was described in detail by Giotakis and coauthors [18].

#### Delineation of tissue compartments and elimination of artifacts

For compartment analysis, tumor cell clusters and stroma in patients with OPSCC as well as the epithelial layer and LP in control patients were manually outlined as subregions using the software function ‘create region of interest’. Tumor cell clusters were defined as areas with visible expression of cytokeratin, possibly including connective tissue cells and immune cells, surrounded by a few µm of stroma. The rest of the stroma was titled stroma. Epithelial layer and LP in control patients were sharply outlined. Regions with artifacts, debris, and air bubbles were visually identified, manually outlined, and excluded.

#### Nuclear, cytoplasm and background segmentation

Segmentation is the creation of content-related regions by combining neighboring pixels that followed a criterion of homogeneity. First, nuclei were segmented and defined as events, the basic units of image analysis. Starting from the nucleus, the cytoplasmic areas belonging to a nucleus were defined in a further segmentation step and assigned to the same event. Nuclear and cytoplasm segmentation were described in detail here [18]. In the current study, precision of nuclear dimensions was ensured by excluding area sizes smaller than 40 µm^2^ and larger than 120 µm^2^. Here, tumor cells were investigated, which in general have larger nuclei than normal cells [29]. Therefore, the larger nuclear dimensions were modified from 100 µm^2^ to 120 µm^2^. Background segmentation was defined in an area free of nuclei. It was analyzed in each tissue sample after setting the nuclear segmentation parameters to 0 [18]. After background segmentation, multiple small events were recognized, corresponding to pixels of the background area.

#### Raw data of immunofluorescence multiplex image cytometry

After segmentation, the software provided the mean of the pixel fluorescence intensities (= mean fluorescence intensity) of each event in each of the four channels, i.e., AF488 for cytokeratin, eFluor570 for vimentin, AF594 for CD45/CD18 and DAPI for the nucleus. The number of the events and the mean fluorescence intensity per event were the outcomes of immunofluorescence multiplex image cytometry. The Raw Data, which were extracted from StrataQuest (TissueGnostics), included the mean fluorescence intensity of each event for each channel, and each tissue compartment in patients with OPSCC and control patients.

### Data analysis

All Raw Data were extracted into the SPSS 26.0 statistic package (SPSS Inc., Chicago, Illinois, USA). To reduce file size, a random sample of 15% of the imported events was drawn. Cell count and cell density were tested for normal distribution and accordingly tested with non-parametrical analysis. For all scanned samples, background mean fluorescence intensities were subtracted from the events’ mean fluorescence intensities for each channel to produce the background corrected mean intensities. If an event’s mean fluorescence intensity was lower than the background mean fluorescence intensity, then the value of the background corrected mean intensity was set to value 1 to prevent negative intensities.

For positive controls and isotypes, cytokeratin was used as a calibration marker for the AF488 channel, vimentin for the eFluor570 channel, and CD45/CD18 for the AF594 channel. It was expected that for each calibration marker, high intensities would be expressed in corresponding positive controls and low intensities in the other biomarkers. It was further expected that low intensities would be expressed in isotype controls for all three channels. In addition, intensities in positive controls were expected to be higher or at least as high as intensities in patient samples, while intensities in isotype controls were expected to be lower or at least as low as intensities in patient samples.

#### Sample exclusion

Obviously flawed samples were excluded. Obvious severe flaws included: (a) patients’ samples or positive controls with background corrected mean intensities in all three channels not above isotype level, (b) positive controls with background corrected mean intensities below the lower 99.9% confidence interval (CI) of the median of patients’ tissue samples, (c) isotype controls with background corrected mean intensities above the lower 99.9% CI of the median of patients’ tissue samples and (d) positive controls, in which the background corrected mean intensities of the calibration-marker was not above the 99.9% CI of the other two biomarkers.

#### Quantile normalization

After background correction and exclusion of samples with obvious severe flaws, the background corrected mean intensities were subjected to channel normalization using the quantile method for high-throughput methods [30]. We used the R script provided by Tang [31]. The effect of the quantile channel normalization is an adjustment of the distribution forms and all statistical position parameters of the three channels. The normalized background corrected mean intensity was titled mean intensity.

#### Definition of cell populations

After the quantile normalization, the sum of the mean intensities of all three channels was calculated and titled “sum intensity”. Sum intensity was classified in 10 deciles (sum-intensity-deciles). For assignments of cells into different cell populations, the mean intensity and sum-intensity-deciles were used. The percentage of the cell populations of each tissue compartments in patients with OPSCC and control patients was the main outcome parameter of the study.

Depending on the expression and co- expression of antibodies, cell populations were defined as anaplastic (no or weak expression of all antibodies), artifacts (intensive expression of all antibodies or simultaneous expression of cytokeratin and CD45/CD18), cells of epithelial differentiation (intensive cytokeratin expression), connective tissue cells (intensive vimentin expression), immune cells (intensive CD45/CD18 with or without vimentin expression), and cells in EMT (intensive vimentin and cytokeratin expression; Table 1).

**Table 1:** Definition of cell populations

Defined cell population	Cell type	Event’s sum-intensity-deciles	Event’s coefficient variation of mean intensity	Event’s mean intensity
Triple-negative	Anaplastic	< 3		
Triple-positive	Artifact	> 5	Cytokeratin, vimentin, CD45/CD18 < 0.2	
Cytokeratin-single-positive	Epithelial or glandular cell			Cytokeratin > 1.2*Mean(Vimentin, CD45/CD18)
Vimentin-single-positive	Fibroblast or endothelial cell			Vimentin > 1.2*Mean(Cytokeratin, CD45/CD18)
CD45/CD18-single-positive	Immune cell			CD45/CD18 > 1.2*Mean(Cytokeratin, vimentin)
Vimentin-CD45/CD18-double-positive	Immune cell		Vimentin, CD45/CD18 < 0.2	Mean(Vimentin, CD45/CD18) > 5*Cytokeratin
Vimentin-cytokeratin-double-positive	Cells in EMT		Cytokeratin, vimentin < 0.2	Mean(Cytokeratin, vimentin) > 5*CD45/CD18
Cytokeratin-CD45/CD18-double-positive	Artifact		Cytokeratin, CD45/CD18 < 0.2	Mean(Cytokeratin, CD45/CD18) > 5*Vimentin

#### Tumor-stroma ratio- desmoplastic reaction

The tumor-stroma ratio was computed by dividing the size of the area of tumor cell clusters by the size of the area of stroma within each OPSCC patient’s tissue.

### Study outcomes

The main outcome of this study was the quantification of the cell populations in patients with HPV + OPSCC and HPV- OPSCC, and control patients, in the different tissue compartments and the whole tissue irrespective of the tissue compartment. Moreover, descriptive statistics were provided for scanned tissue area, total cell count, cell density and nucleus size.

As secondary outcomes, we investigated whether proportions of cell populations were associated to disease severity and prognosis. Disease severity was based on the 8th American Joint Commission on Cancer (AJCC) Staging. Specifically, we examined the association of proportions of cell populations with the tumor (T)-status, nodal (N)-status and stage. Disease prognosis was based on disease-free survival (DFS) status, DFS time (in months), overall survival (OS) status and OS time (in months).

## Results

### Patient population

After image and data analysis, FFPE tissue samples of eight patients with HPV + OPSCC, six patients with HPV- OPSCC, and six control patients were suitable for analysis. Samples from two patients with HPV- OPSCC and two control patients were excluded based on an obvious severe flaw mentioned in the sample exclusion criteria. The background corrected mean intensities of these four samples in all three channels were not above isotype level. This implied that these samples had staining failures or were of poor quality or were biologically implausible.

The mean age ± standard deviation (SD) of the 14 patients with OPSCC was 67.4 ± 9.8 years (y) (range: 48–82 y). Eight out of 14 patients with OPSCC and 4/6 control patients were men, and the mean age was 30.4 ± 6.2 ys (range: 22–39 ys). The most frequent tumor stage according to 8th Edition of the AJCC Staging was IVa (6/14 OPSCC patients). According to histopathologic evaluation, 5/8 HPV + and 4/6 HPV- OPSCC tumor samples were poorly differentiated. Non-keratinizing tumors were documented in 12/14 patients with OPSCC. A basaloid tumor type was noted in two patients with HPV- OPSCC. For all 14 patients, diagnosis was confirmed in 2020 (Tables 2 and 3).

**Table 2:** Clinical data of patients with oropharyngeal squamous cell carcinoma

Case	Age^a^	Gender^b^	Smoking^c^	Alcohol consumption^d^	Comorbidities^e^	Hemoglobin^f^	BMI^g^
1	82	W	0	0	2	144	24
2	64	M	0	0	1	148	25
3	65	W	80	2	3	142	32
4	68	W	0	0	1	145	23
5	58	M	30	2	1	150	23
6	75	W	0	0	2	130	22
7	81	W	0	0	2	121	19
8	75	M	0	0	2	144	24
9	48	M	0	0	1	135	27
10	63	M	35	0	2	144	23
11	64	M	20	0	1	148	20
12	55	W	10	0	1	135	18
13	72	M	50	0	1	141	24
14	74	M	40	1	3	135	33

^a^in years; ^b^W: woman; M: man; ^c^in pack-years; ^d^ (0 = none or normal; 1 = mild; 2 = heavy); ^e^based on ASA (American society of anesthesiologists’ physical status) score (1 = healthy; 2 = mild to moderate systemic disease; 3 = severe non-incapacitating disease process; 4 = severe incapacitating disease process; 5 = moribund patients; 6 = declared brain-dead); ^f^before treatment (g/dl); ^g^body-mass index

**Table 3:** Pathological, treatment and follow up data of patients with oropharyngeal squamous cell carcinoma

Case	T^a^	N^b^	M^c^	P16	AJCC^d^	Diagnosis^e^	Primary treatment	DFS^f^	DFS time^g^	OS^h^	OS time^g^	Follow-up^i^
1	2	2b	0	(-)	IVa	05/20	CRTH^j^	1	30	1	34	CR^m^
2	1	1	0	(+)	I	08/20	OP^k^/adj. RT^l^	1	26	1	31	CR
3	4a	2b	0	(-)	IVa	04/18	OP/adj. RT	1	53	1	59	CR
4	1	2b	0	(-)	IVa	04/20	CRTH	1	28	1	35	CR
5	2	2b	0	(-)	IVa	09/20	OP/adj. CRTH	0	5	1	30	PR^n^
6	2	0	0	(+)	I	09/20	OP	1	28	1	28	CR
7	4	1	0	(+)	III	04/20	CRTH	1	33	1	35	CR
8	2	2b	0	(-)	IVa	05/20	CRTH	1	29	1	34	CR
9	3	2	0	(+)	II	05/20	CRTH	1	29	1	34	CR
10	2	1	0	(+)	I	06/20	CRTH	1	28	1	33	CR
11	4	2	0	(+)	III	10/20	CRTH	1	24	1	29	CR
12	3	2	0	(+)	II	08/20	CRTH	1	23	1	31	CR
13	2	1	0	(+)	I	08/20	CRTH	1	23	1	31	CR
14	2	2c	0	(-)	IVa	08/20	CRTH	1	23	1	31	CR

^a^Tumor (clinical stage); ^b^Nodes (clinical stage); ^c^Metastasis; ^d^American Joint Commission on Cancer stage, 8th edition; ^e^Month/year; ^f^Disease-free survival event (0 = recurrency, 1 = no recurrency); ^g^in months; ^h^Overall survival event (0 = death, 1 = alive); ^i^data until April 2023; ^j^Chemoradiotherapy; ^k^Operation; ^l^Radiotherapy; ^m^Complete remission; ^n^Partial remission

### Tissue area, cell count, cell density and nucleus size

The scanned area, cell count, and cell density per OPSCC and control sample were not normally distributed (all p < 0.05). The median scanned area per OPSCC and control sample was 7.6 mm^2^ (lower quartile 3.4 mm^2^ to upper quartile 13.4 mm^2^). Out of 4,300,000 cells total, a median of 32,133 cells (lower quartile 12,633 cells to upper quartile 70,980 cells) were recognized per OPSCC and control sample after selecting the 15% sample. Although the scanned area was larger in OPSCC than in control samples (Mann-Whitney Test: all p < 0.020), the median cell density in OPSCC and control samples (4,613 cells/mm^2^, lower quartile 3,120 cells/mm^2^ to upper quartile 6,101 cells/mm^2^) did not differ significantly (Mann-Whitney Test; all p > 0.188). No significant differences were observed for nucleus size between patients with OPSCC and control patients, but compartmental distribution differed (Table 4). Between HPV + and HPV- OPSCC samples, no significant differences in scanned total area, cell count and cell density were noted (Mann-Whitney Test; all p > 0.060).

**Table 4:** Scanned area and nucleus size in OPSCC samples and controls

Parameter	OPSCC			Controls		
	Tumor cell cluster	Stroma	Whole sample	Epithelial area	Lamina propria	Whole sample
Scanned area^a^ (mm^2^)	6.9 (4.4–11.6)	6.1 (2.4–18.1)^b^		1.8 (0.6–4.4)	3.5 (0.7–8.6)^c^	
Nucleus size^a^ (µm^2^)			67 (53–88)			68 (55–85)

a. median value (lower to upper quartile)

b. No significant differences were observed between tumor cell clusters and stroma in OPSCC samples (p > 0.2; Wilcoxon paired samples)

c. No significant differences were observed between epithelial area and lamina propria in controls (p > 0.2; Wilcoxon paired samples)

### Cell populations

#### Anaplastic cells in patients with HPV + OPSCC, patients with HPV- OPSCC, and control patients

**Fig. 1:**
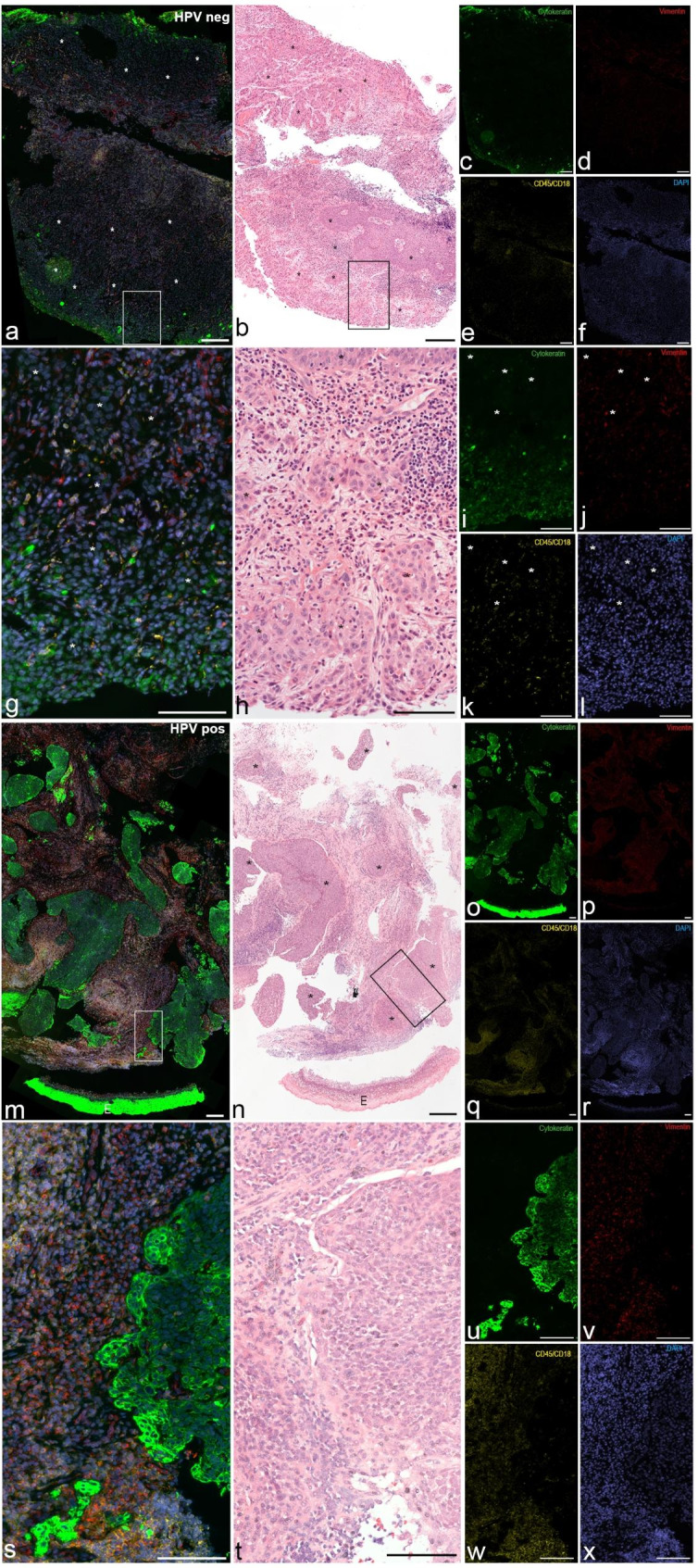
**Comparison between patients with HPV negative and HPV positive oropharyngeal squamous cell carcinoma**. Images of oropharyngeal squamous cell carcinoma (OPSCC) in an HPV negative (**a-l**) and an HPV positive (**m-x**) patient. Immunofluorescent triple stained overlays are shown in **a**, **g**, **m**, **s** and single channel fluorescent stains are presented in **c-f**, **i-l**, **o-r** and **u-x**. Corresponding hematoxylin-eosin stained sections are presented in **b**, **h**, **n**, **t**. Framed areas indicate high magnification images below. Scale bars **a-f** & **m-r** 200 μm, **g-l** & **s-x** 100 μm

Anaplastic cells were encountered more frequently in patients with HPV- OPSCC (21%) than in patients with HPV + OPSCC (6%; Table 5; Fig. 1). Fewer anaplastic cells were encountered in the control patients (2%) than in patients with OPSCC (15%; Table 5).

**Table 5:** Cell populations in patients with oropharyngeal squamous cell carcinoma (OPSCC) and in control patients

Cell populations^a^ and comparison^b^	Tumor cell cluster/epithelial layer			Stroma/lamina propria			Irrespective of compartment		
	OPSCC		Controls	OPSCC		Controls	OPSCC		Controls
	HPV+	HPV-		HPV+	HPV-		HPV+	HPV-	
	(A)	(B)	(C)	(A)	(B)	(C)	(A)	(B)	(C)
Triple-negative	1.5%	10.6%	0.0%	11.4%	24.7%	3.8%	5.5%	21.4%	1.7%
*Anaplastic cells*	C	A C		C	A C		C	A C	
Triple-positive	0.6%	0.3%	0.6%	0.6%	0.3%	0.7%	0.6%	0.3%	0.6%
*Artifacts*	B		B	B		B	B		B
Cytokeratin-single-positive	55.0%	44.4%	75.4%	16.8%	11.3%	28.9%	39.5%	19.2%	50.6%
*Cells of epithelial differentiation*	B		A B	B		A B	B		A B
Vimentin-single-positive	17.8%	26.4%	2.5%	39.8%	30.3%	16.8%	26.7%	29.4%	9.1%
*Connective tissue cells*	C	A C		B C	C		C	A C	
CD45/CD18-single-positive	21.1%	15.9%	19.8%	27.2%	28.1%	33.6%	23.6%	25.2%	28.1%
*Immune cells*	B C		B		A	A B		A	A B
Vimentin-CD45/CD18-double-positive	3.7%	2.1%	0.8%	4.1%	4.9%	15.6%	3.9%	4.3%	9.2%
*Immune cells*	A C	C			A	A B		A	A B
Vimentin-cytokeratin-double-positive	0.2%	0.3%	0.0%	0.1%	0.1%	0.2%	0.1%	0.3%	0.3%
*Cells in EMT*	C	C				A		A	A
Cytokeratin-CD45/CD18-double-positive	0.2%	0.0%	0.7%	0.1%	0.1%	0.1%	0.2%	0.0%	0.4%
*Artifacts*	B		A B				B		A B

a. Column percentage of all cells per compartment

b. Results were based on two-sided tests. For each significant pair, the key of the category with the smaller column proportion appeared in the category with the larger column proportion. Significance level for upper case letters (A, B, C) was 0.05. Tests were adjusted for all pairwise comparisons within a row of each innermost subtable using the Bonferroni correction

#### Tumor cell clusters in patients with HPV + OPSCC and patients with HPV- OPSCC

In tumor cell clusters of HPV + OPSCC samples, higher proportions of cells of epithelial and immune differentiation and lower proportions of connective tissue cells were encountered than in HPV- OPSCC samples (55% vs. 44%, 25% vs. 18%, and 18% vs. 26%, respectively; Table 5; Fig. 1).

#### Stroma in patients with HPV + OPSCC and patients with HPV- OPSCC

In stroma, higher proportions of cells of epithelial and connective tissue differentiation were found in patients with HPV + OPSCC (17% and 40%, respectively) compared to those in patients with HPV- OPSCC (11% and 30%, respectively). A similar number of immune cells was found in patients with HPV + OPSCC and patients with HPV- OPSCC (31% and 33%, respectively; Table 5; Fig. 1).

#### Comparison of control patients with patients with OPSCC

**Fig. 2:**
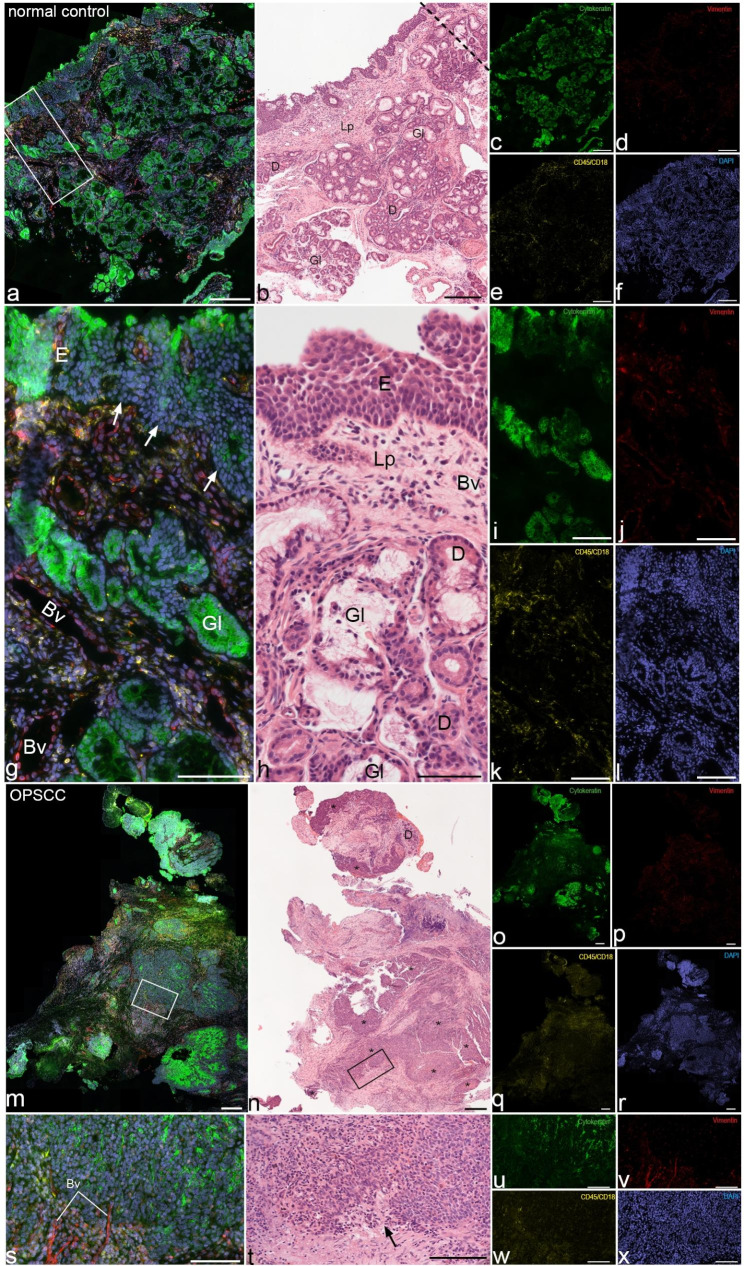
**Comparison between patients with oropharyngeal squamous cell carcinoma and control patients**. Images of normal oropharyngeal tissue (**a-l**) and oropharyngeal squamous cell carcinoma (OPSCC) (**m-x**). Immunofluorescent triple stained overlays are shown in **a**, **g**, **m**, **s** and single channel fluorescent stains are presented in **c-f**, **i-l**, **o-r** and **u-x**. Corresponding hematoxylin-eosin stained sections are presented in **b**, **h**, **n**, **t**. Framed areas indicate high magnification images below and is indicated by dashed lines in **b**. Arrow in **t** indicates “unsharp” tissue borders between connective and overlying epithelial layers. Scale bars **a-f** & **m-r** 200 μm, **g-l** & **s-x** 100 μm. Bv blood vessel, D ducts of submucosal glands, E mucosal epithelium, Gl submucosal glands, Lp lamina propria

In control samples, the proportions of cells of epithelial, connective tissue and immune differentiation were 53%, 10% and 27%, respectively, compared to 29%, 28% and 28% of the patients with OPSCC (Table 5; Fig. 2). In OPSCC and control samples, the proportions of cells in EMT were less than 1% (Table 5).

#### Comparison of cell populations between the epithelial layer and lamina propria

In control patients, proportions of epithelial cells in epithelial layer exceeded proportions of glandular cells in LP (75% vs. 29%, respectively). More connective tissue cells were found in LP than in epithelial layer (17% vs. 3%, respectively). Fewer immune cells were encountered in epithelial layer than in LP (21% vs. 49%, respectively; Table 5; Fig. 2).

In patients with OPSCC, proportions of cells of epithelial differentiation were higher in tumor cells clusters than in stroma (52% vs. 13%, respectively). More connective tissue cells were found in stroma than in tumor cell clusters (34% vs. 20%, respectively). Fewer immune cells were encountered in tumor cell clusters than in stroma (23% vs. 32%, respectively; Table 5; Fig. 2).

#### Fluorescence intensity and cell populations in positive – and isotype controls

**Fig. 3:**
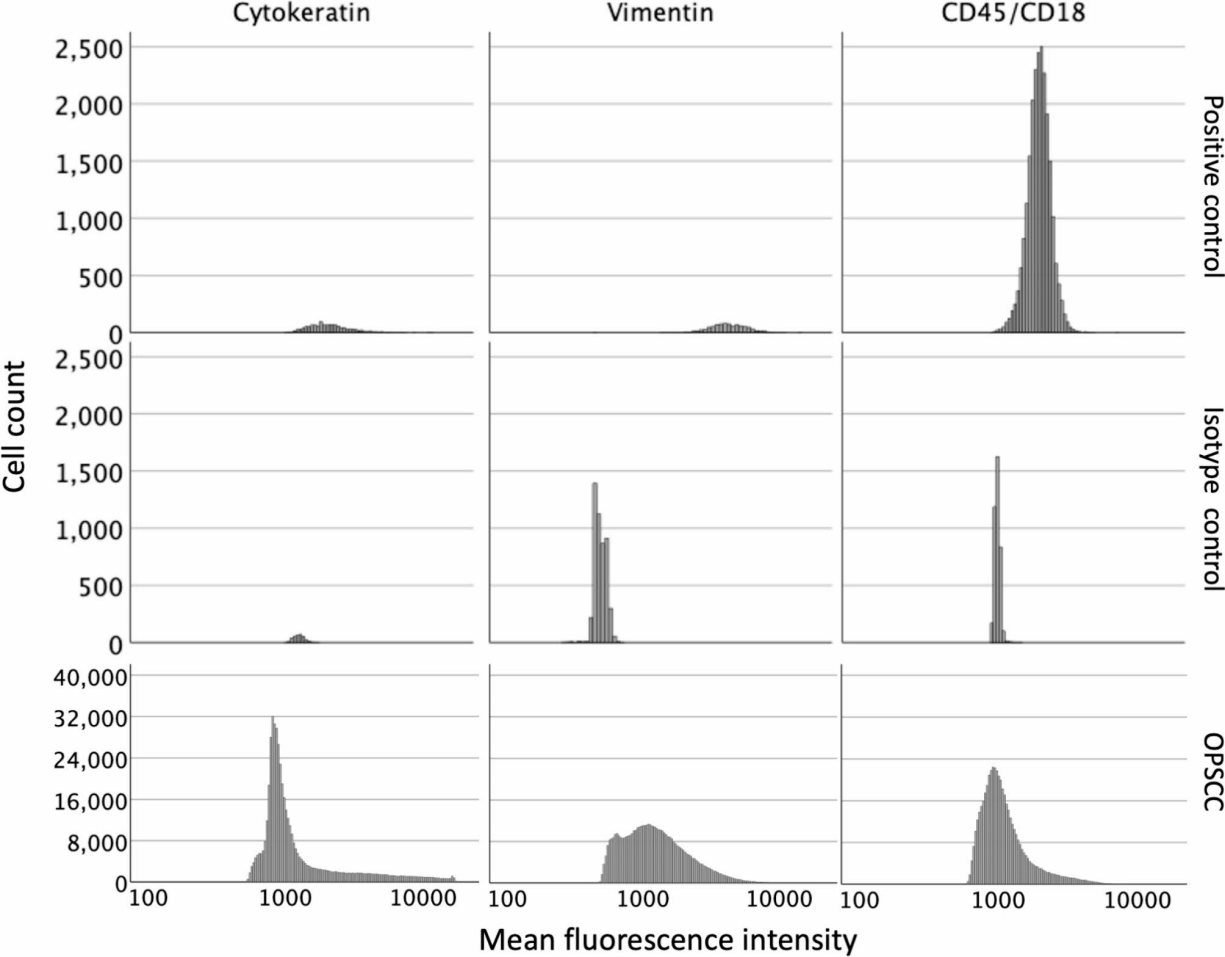
**Histograms of positive- and isotype controls, and patients with oropharyngeal squamous cell carcinoma positive controls**. Comparison of histograms of fluorescence signals between positive controls, patients with oropharyngeal squamous cell carcinoma (OPSCC) and isotype controls. Histograms of fluorescence signals after control incubation in the middle histograms (isotype controls) and specific fluorescence signals after test incubation in the upper (positive controls) and lower (patients with OPSCC) histograms. Presentation of a random cell sample of the positive controls, their isotype controls and the patients with OPSCC. X-axis: mean intensity in logarithmic scale. Y-axis: Cell count. The left histograms represent cytokeratin fluorescence signal of CAL-27 tumor cell line, its isotype and patients with OPSCC, the middle histograms vimentin fluorescence signal of human gingiva fibroblast cell line, its isotype and patients with OPSCC and the right histograms CD45/CD18 fluorescence signal of lymphoma tissue, its isotype and patients with OPSCC

The cytokeratin mean fluorescence intensity (median ± SD) in tumor cell line was 2,447 ± 1,120 and in its isotype, it was 1,348 ± 122. The vimentin mean fluorescence intensity in fibroblast cell line was 4,504 ± 1,574 and in its isotype, it was 493 ± 50. The CD45/CD18 mean fluorescence intensity in lymphoma was 2,202 ± 441 and in its isotype, it was 1,070 ± 50 (Fig. 3).

After applying background correction and normalization, all cells in cytokeratin-positive-controls were either cells of epithelial differentiation (63%) or anaplastic (34%). In cytokeratin-isotype, 100% of the cells were triple-negative. In fibroblast-positive-control, all cells were either connective tissue cells (87%) or CD45/CD18-vimentin-double-positive (12%). In fibroblast-isotype, most cells were triple-negative (95%). In CD45/CD18-positive-controls, most cells were immune cells (71%; i.e., CD45/CD18-single positive cells were 50% and CD45/CD18-vimentin-double-positive cells were 21%) or anaplastic (26%). In CD45/CD18-isotype, all cells were triple-negative (100%).

### Association of proportions of cell populations with disease severity and prognosis

#### Irrespective of the tissue compartment

Proportions of cell populations in patients with OPSCC did not differ significantly between the different sizes and extents of the main tumor (T-status; Kruskal Wallis test; all p > 0.085), regional nodal involvement (N-status; all p > 0.11) and DFS status (all p > 0.15). Proportions of cell populations did not correlate with DFS-time (all p > 0.11). No patient passed away during follow-up.

Proportions of cells of epithelial differentiation were two to three times larger in patients with stage II (mean value: 45%) and III (49%) compared to patients with stage I (17%) and IV (21%; Kruskal Wallis test; p = 0.048). Proportions of the rest cell populations did not differ significantly between the different stages (p > 0.2).

#### Tumor cell clusters

In tumor cell clusters, proportions of cell populations in patients with OPSCC did not differ significantly between the different sizes and extents of the main tumor (T-status; all p > 0.15), regional nodal involvement (N-status; all p > 0.051), stage (all p > 0.14) and DFS status (all p > 0.2). Proportions of cell populations did not correlate with DFS-time (all p > 0.2).

#### Stroma

In stroma, proportions of cell populations in patients with OPSCC did not differ significantly between the different sizes and extents of the main tumor (T-status; all p > 0.10), regional nodal involvement (N-status; all p > 0.087), stage (all p > 0.092) and DFS status (all p > 0.15). Proportions of cell populations did not correlate with DFS-time (all p > 0.067).

### Tumor-stroma ratio

The ratio of tumor cell cluster to stroma was not normally distributed (p < 0.001). The median ratio of tumor cell cluster to stroma was smaller in patients with HPV- OPSCC (median 0.45; lower quartile 0.22 to upper quartile 0.60) than in patients with HPV + OPSCC (median 1.55; lower quartile 0.97 to upper quartile 10.3; Mann-Whitney Test p = 0.050; Fig. 1). This implied that larger areas of tumor cell clusters were observed in HPV + OPSCC samples compared to HPV- OPSCC samples, while smaller areas of stroma were observed in HPV + OPSCC samples compared to HPV- OPSCC samples.

For a detailed description of immunohistochemical details on surface marker expression in OPSCC and control samples please refer to Additional file 1.

## Discussion

In this study, a novel four-channel immunofluorescence microscopy technique for the simultaneous detection of three antibodies and DAPI in FFPE tissue was applied for cellular profiling of the OPSCC tissue and microenvironment. Descriptive data on proportional variations of different cell populations and tissue subunits, depending on the HPV status in OPSCC, were provided in a whole slide analysis.

Major advantages of this study are worth mentioned. One important aspect was the whole slide analysis. This overcomes difficulties encountered in immunohistopathological evaluation of OPSCC. Macroscopically, the tumor samples might be small, damaged, and contain many necrotic areas which makes them difficult to examine. Microscopically, the tumor cells might have lost their differentiation characteristics. During conventional semi-quantitative examinations of histopathological tissue sections, the examiner might be inclined to select only high-power fields with well-preserved and easily interpretable tissue areas. Image cytometry allows for larger, three-dimensional areas of moderate sample quality to be evaluated. Although this increases the probability of errors, higher cell numbers are evaluated and representativeness is improved.

In addition, the technique preserves tissue architecture, as opposed to the isolation of cells in flow cytometry. One further advantage of immunofluorescence multiplex image cytometry is high throughput evaluation on commonly available FFPE samples. In particular, all three antibodies are expressed in the same tissue sample in contrast to immunohistochemistry and multiple studies that have examined cells of epithelial, connective tissue and immune differentiation, separately from each other [9, 15, 32–46]. Moreover, plausibility of cell selection criteria was confirmed through backward visualization. Furthermore, fluorophores are more channel specific than dyes and comparably quantifiable. Also, the intensity of direct immunofluorescence correlated with the protein concentration of interest, although this correlation is often not linear [47].

Moreover, we managed to quantify the de-differentiation of the tumor, the desmoplastic reaction and the antitumoral host immune response, which may allow for comparison within and/or between studies in the future. This was additionally a study with a control group. Control groups reduce bias by allowing researchers to confirm that study results are due to the manipulation of independent variables rather than extraneous variables [48].

Finally, analysis of different compartments, e.g., tumor cell cluster and stroma in OPSCC, was feasible. Tumor cell clusters were intentionally defined to include a small amount of the surrounding stroma. Known as the desmoplastic reaction, this interaction is an important mediator of cancer invasion and metastasis [49]. Demarcation of tumor cell clusters can be particularly difficult in poorly differentiated OPSCC due to the presence of numerous small tumor cell clusters as opposed to cohesive tumors with clearly outlined margins [50]. Our strategy allowed for exact definition of tumor cell clusters in all FFPE tissue samples, regardless of their level of cohesiveness.

On the contrary, data analysis was an elaborate process, despite consisting mainly of background correction, quantile normalization, and definition of cell populations. As no background correction tools were available from StrataQuest, background intensities had to be subtracted from the event intensities for each channel. Resulting negative fluorescence intensities were set to one. All associated event intensities less than zero after background correction were considered invalid, as negative signal-background relation lacks physiological sense.

The background corrected mean intensities substantially differed between channels. These variations occurred mainly due to differences in binding affinities and fluorophore loads of the antibodies, as well as characteristics of the channel filters. Out of several available methods for normalization, well balanced channel intensities were only achieved through quantile normalization.

Cell populations were defined by their mean intensities per channel. Intensity cutoffs based on the positive and isotype controls were not applicable due to overlaps and substantial differences within the controls. Instead, sum-intensity-deciles, coefficient of variation of mean intensities and ratios between channel intensities were applied for statistical analysis (Table 1). However, investigator-dependent issues may still exist.

The main outcome of the study was the percentage of the cell populations of each tissue compartments in patients with OPSCC and control patients. This was based on the antibodies that were applied in this study. These sufficiently recognized leukocytes, endothelial cells, fibroblasts and smooth muscle cells. Most of the common cells of epithelial differentiation presenting in OPSCC were identified, i.e., human cytokeratin 4–6, 8, 10, 13 and 18 types. Types 7 and 19 were not recognized [32].

Results allowed for significant observations. In OPSCC patients, about 29% of all cells expressed an epithelial differentiation, 28% a predominantly connective tissue differentiation, and 28% a leukocytic differentiation. About 15% of all cells could not be assigned to any of these three cell populations. A much smaller percentage of anaplastic cells (below 2%) was encountered in control patients, in which about 51% of all cell expressed an epithelial differentiation, 9% a predominantly connective tissue differentiation, and about 37% a leukocytic differentiation. The direct vicinity of the control samples to the palatine tonsils, an area with highly so-called physiological inflammation, may explain the high proportion of immune cells in the control patients. Immune cells were found in the LP (49%), but also in the epithelial layer (22%). They physiologically transmigrate the epithelial layer and thus enter the lumen of the upper aerodigestive tract. Chronic snoring might further increase the proportion of inflammatory cells in oropharyngeal mucosa.

The results in the control patients suggested that in normal oropharyngeal mucosa very few cells (below 2%) cannot be assigned to one of the three basic cell populations in contrast to the higher number of anaplastic cells observed in OPSCC patients (15%). The de-differentiation of the tumor cells might explain this observation. Assumably, the differentiating features examined in patients with OPSCC can no longer be detected. In line with this observation, we noted that the cytokeratin mean intensity of the poorly differentiated tumor cell line CAL-27 was lower than that of the epithelial cells of normal mucosa. This might indicate that poorly differentiated tumor cell cluster areas with low or isotype-similar cytokeratin expression were not defined as tumor cell clusters during delineation of tissue compartments. Therefore, it cannot be ruled out that some tumor cell cluster areas were falsely classified as stroma. While the results of the whole tissue irrespective of compartment appear to be quite reliable, the results of the subdivision into the individual compartments such as tumor cell clusters and stroma as well as the results of tumor-stroma ratio should be interpreted with caution.

The abundance of cell populations was in line with existing data. In particular, the higher percentage of cells of epithelial differentiation in tumor cell clusters in HPV + samples compared to HPV- OPSCC samples (Table 5) indicated a higher degree of differentiation in HPV + OPSCC samples, which was in line with recent data [15, 35, 36]. The lower percentage of connective tissue cells in tumor cell clusters of patients with HPV + OPSCC (Table 5) may be associated with a lower fibroblast response compared to patients with HPV-OPSCC. This observed “desmoplastic reaction” supports cancer migration and invasion in vitro, as reported by Bolt [9] and Rahrotaban [37]. In contrast, a higher percentage of connective tissue cells was identified in the stroma of HPV + compared to HPV- OPSCC samples (Table 5), as described by Mohamed and coauthors [38]. Furthermore, we observed a higher number of immune cells in tumor cell clusters of HPV + OPSCC samples compared to HPV- samples (Table 5). Increased recruitment of immune cells and tumor infiltrating lymphocytes in patients with HPV + OPSCC compared to patients with HPV- OPSCC has been reported in several studies [39–44].

Antibodies’ co-expression patterns resulted in identification of cytokeratin-vimentin-double-positive cells, which could be cells undergoing epithelial-mesenchymal transition (EMT) [51]. The percentage of cells in EMT in tumor cell clusters of patients with OPSCC was two (0.2%; HPV+) to three (0.3%; HPV-) times larger than the percentage of cells in EMT in control samples (0.0%; Table 5). Until now, no significant differences in EMT expression depending on the HPV status have been reported [45, 46]. Nevertheless, more cells in EMT may have been detected, if additional staining for other EMT markers, such as E-cadherin and β-catenin had been performed [45, 46].

One additional outcome of the study was the tumor-stroma ratio. A strong desmoplastic reaction with small, scattered tumor cell clusters was more often observed in HPV- OPSCC samples than in HPV + OPSCC samples (Fig. 1). Comparable data are scarce. These findings may partially explain the better outcomes observed in HPV + OPSCC. Better outcomes of head and neck malignancies with big tumor cell clusters and little desmoplastic reaction have been reported [52], whereas abundant stroma and small tumor cell clusters were associated with adverse prognostic factors [53].

This study had limitations. A major limitation was the elaborate nature of all steps of immunofluorescence multiplex image cytometry, from sample collection to statistical analysis. Only acquisition of the scanned tissue area, including positive controls, isotype controls and channel-spillover tissue (approximately 750 mm^2^) required more than 100 working hours [18]. This meticulous workup and the limited funding allowed examination of only 20 patients. Thus, investigation of the correlation of the cell populations’ relative proportions with disease prognosis was not justified. Statistics were based on the number of the recognized cells (4,300,000 cells in total). Furthermore, the follow-up time was insufficient for important conclusions (mean overall survival time: 34 months). This case number and the follow-up time were too limited to draw a solid conclusion. Therefore, the results of the current study might lack clinical significance. However, its potential applications in more clinically relevant areas of research may compensate for these study’s deficits in clinical significance.

A second limitation of this study was the lack of additional investigations due to the limited funding, such as the genetic background of these samples, by examination of the mutations or copy number variations of driver genes E6 and E7 [54]. Moreover, it would be interesting to investigate the HPV-status in control patients.

A third limitation included the dependence of the identification of tumor cell areas from high cytokeratin expression. This might have led to missing tumor cell clusters areas that were more de-differentiated. Future similar studies should verify and cross-check the identification of tumor cell areas in hematoxylin and eosin stains.

A fourth limitation included the absence of characterization and differentiation between the different types of immune cells, such as neutrophils, eosinophils, T-cells, B-cells, natural killer cells or macrophages. However, this would necessitate a different study design, with advanced hard- and/or software, much more additional fluorescence channels, as well as search, selection and titration of different antibodies to identify these immune cell types.

## Conclusions

This preliminary report provided an analysis of proportions of cells of epithelial, connective tissue and immune differentiation in FFPE tissue samples of patients with HPV + and HPV- OPSCC, as well as control samples, using whole-slide immunofluorescence multiplex image cytometry. De-differentiation of the tumor cells was quantified. De-differentiation was higher in HPV- OPSCC samples than in HPV + OPSCC samples. In tumor cells clusters, the antitumoral host immune response was higher in HPV + OPSCC than in HPV- OPSCC samples, whereas the fibroblast response was higher in HPV- OPSCC than in HPV + OPSCC samples. A more intensive desmoplastic reaction with scattering of tumor cell clusters was observed in HPV- OPSCC samples. Future studies may focus on the correlation of these findings with disease prognosis, examine the distribution of different immune cells such as neutrophils, eosinophils and lymphocytes, and include markers of EMT.

### Electronic supplementary material

Below is the link to the electronic supplementary material.

Supplementary Material 1

## Data Availability

The datasets used and/or analysed during the current study are available from the corresponding author on reasonable request.

## List of Abbreviations

OPSCC: Oropharyngeal squamous cell carcinomas
HPV: Human papilloma virus
HPV+: HPV positive
HPV-: HPV negative
FFPE: Formalin-fixed paraffin embedded
LP: Lamina propria
hGF: Human gingival fibroblast
DAPI: 4’,6-Diamidin-2-phenylindole
AF488: Alexa fluor 488
AF594: Alexa fluor 594
AJCC: American Joint Commission on Cancer
DFS: Disease-free survival
OS: Overall survival
ASA: American society of anesthesiologists’ physical status

## References

[1] Pezzuto F, Buonaguro L, Caponigro F, Ionna F, Starita N, Annunziata C, Buonaguro FM, Tornesello ML (2015). Update on Head and Neck Cancer: current knowledge on epidemiology, risk factors, molecular features and Novel Therapies. Oncology.

[2] van Monsjou HS, van Velthuysen ML, van den Brekel MW, Jordanova ES, Melief CJ, Balm AJ (2012). Human papillomavirus status in young patients with head and neck squamous cell carcinoma. Int J Cancer.

[3] Ang KK, Harris J, Wheeler R, Weber R, Rosenthal DI, Nguyen-Tan PF, Westra WH, Chung CH, Jordan RC, Lu C (2010). Human papillomavirus and survival of patients with oropharyngeal cancer. N Engl J Med.

[4] Licitra L, Perrone F, Bossi P, Suardi S, Mariani L, Artusi R, Oggionni M, Rossini C, Cantu G, Squadrelli M (2006). High-risk human papillomavirus affects prognosis in patients with surgically treated oropharyngeal squamous cell carcinoma. J Clin Oncol.

[5] Rietbergen MM, Brakenhoff RH, Bloemena E, Witte BI, Snijders PJ, Heideman DA, Boon D, Koljenovic S, Baatenburg-de Jong RJ, Leemans CR (2013). Human papillomavirus detection and comorbidity: critical issues in selection of patients with oropharyngeal cancer for treatment de-escalation trials. Ann Oncol.

[6] Wansom D, Light E, Worden F, Prince M, Urba S, Chepeha DB, Cordell K, Eisbruch A, Taylor J, D’Silva N (2010). Correlation of cellular immunity with human papillomavirus 16 status and outcome in patients with advanced oropharyngeal cancer. Arch Otolaryngol Head Neck Surg.

[7] Almangush A, Mäkitie AA, Triantafyllou A, de Bree R, Strojan P, Rinaldo A, Hernandez-Prera JC, Suárez C, Kowalski LP, Ferlito A (2020). Staging and grading of oral squamous cell carcinoma: an update. Oral Oncol.

[8] De Wever O, Demetter P, Mareel M, Bracke M (2008). Stromal myofibroblasts are drivers of invasive cancer growth. Int J Cancer.

[9] Bolt R, Foran B, Murdoch C, Lambert DW, Thomas S, Hunter KD (2018). HPV-negative, but not HPV-positive, oropharyngeal carcinomas induce fibroblasts to support tumour invasion through micro-environmental release of HGF and IL-6. Carcinogenesis.

[10] Whiteside TL (2008). The tumor microenvironment and its role in promoting tumor growth. Oncogene.

[11] Senovilla L, Vacchelli E, Galon J, Adjemian S, Eggermont A, Fridman WH, Sautes-Fridman C, Ma Y, Tartour E, Zitvogel L (2012). Trial watch: prognostic and predictive value of the immune infiltrate in cancer. Oncoimmunology.

[12] Fridman WH, Pages F, Sautes-Fridman C, Galon J (2012). The immune contexture in human tumours: impact on clinical outcome. Nat Rev Cancer.

[13] Chu PG, Weiss LM (2002). Keratin expression in human tissues and neoplasms. Histopathology.

[14] Moll R, Franke WW, Schiller DL, Geiger B, Krepler R (1982). The catalog of human cytokeratins: patterns of expression in normal epithelia, tumors and cultured cells. Cell.

[15] Woods RSR, Keegan H, White C, Tewari P, Toner M, Kennedy S, O’Regan EM, Martin CM, Timon CVI, O’Leary JJ (2017). Cytokeratin 7 in Oropharyngeal squamous cell carcinoma: a junctional biomarker for human papillomavirus-related tumors. Cancer Epidemiol Biomarkers Prev.

[16] Morbini P, Capello GL, Alberizzi P, Benazzo M, Paglino C, Comoli P, Pedrazzoli P (2015). Markers of squamocolumnar junction cells in normal tonsils and oropharyngeal cancer with and without HPV infection. Histol Histopathol.

[17] Regauer S, Beham A, Mannweiler S (2000). CK7 expression in carcinomas of the Waldeyer’s ring area. Hum Pathol.

[18] Giotakis AI, Dudas J, Glueckert R, Dejaco D, Ingruber J, Fleischer F, Innerhofer V, Pinggera L, Bektic-Tadic L, Gabriel SAM (2021). Characterization of epithelial cells, connective tissue cells and immune cells in human upper airway mucosa by immunofluorescence multichannel image cytometry: a pilot study. Histochem Cell Biol.

[19] Ecker RC, Rogojanu R, Streit M, Oesterreicher K, Steiner GE (2006). An improved method for discrimination of cell populations in tissue sections using microscopy-based multicolor tissue cytometry. Cytometry A.

[20] Ecker RC, Steiner GE (2004). Microscopy-based multicolor tissue cytometry at the single-cell level. Cytometry A.

[21] Healy S, McMahon J, Owens P, Dockery P, FitzGerald U (2018). Threshold-based segmentation of fluorescent and chromogenic images of microglia, astrocytes and oligodendrocytes in FIJI. J Neurosci Methods.

[22] Zanoni DK, Patel SG, Shah JP. Changes in the 8th Edition of the american Joint Committee on Cancer (AJCC) staging of Head and Neck Cancer: Rationale and Implications. Curr Oncol Rep 2019, 21(6). 10.1007/s11912-019-0799-x PMC6528815 30997577

[23] Runge A, Vales A, Pommer G, Raab H, Prossliner H, Randhawa A, Schennach H, Riechelmann H (2023). Perioperative Blood Transfusion in Head and Neck Cancer Revisited. Laryngoscope.

[24] Lydiatt WM, Patel SG, O’Sullivan B, Brandwein MS, Ridge JA, Migliacci JC, Loomis AM, Shah JP (2017). Head and Neck cancers-major changes in the american Joint Committee on cancer eighth edition cancer staging manual. CA Cancer J Clin.

[25] Jiang L, Ji N, Zhou Y, Li J, Liu X, Wang Z, Chen Q, Zeng X (2009). CAL 27 is an oral adenosquamous carcinoma cell line. Oral Oncol.

[26] Lechner M, Schartinger VH, Steele CD, Nei WL, Ooft ML, Schreiber LM, Pipinikas CP, Chung GT, Chan YY, Wu F (2021). Somatostatin receptor 2 expression in nasopharyngeal cancer is induced by Epstein Barr virus infection: impact on prognosis, imaging and therapy. Nat Commun.

[27] Mahanonda R, Sa-Ard-Iam N, Montreekachon P, Pimkhaokham A, Yongvanichit K, Fukuda MM, Pichyangkul S (2007). IL-8 and IDO expression by human gingival fibroblasts via TLRs. J Immunol.

[28] Tarnowski BI, Spinale FG, Nicholson JH (1991). DAPI as a useful stain for nuclear quantitation. Biotech Histochem.

[29] Baba AI, Câtoi C. Tumor cell morphology. Comparative oncology. edn.: The Publishing House of the Romanian Academy; 2007. 20806453

[30] Bolstad BM, Irizarry RA, Astrand M, Speed TP (2003). A comparison of normalization methods for high density oligonucleotide array data based on variance and bias. Bioinformatics.

[31] Gibbons MD, Manne U, Carroll WR, Peters GE, Weiss HL, Grizzle WE (2001). Molecular differences in mucoepidermoid carcinoma and adenoid cystic carcinoma of the major salivary glands. Laryngoscope.

[32] Channir HI, Kiss K, Rubek N, Andersen J, Georgsen JB, Rathje GS, Charabi BW, von Buchwald C, Lajer CB (2018). Comparison of clinical, radiological and morphological features including the distribution of HPV E6/E7 oncogenes in resection specimens of oropharyngeal squamous cell carcinoma. Oral Oncol.

[33] Tosi A, Parisatto B, Menegaldo A, Spinato G, Guido M, Del Mistro A, Bussani R, Zanconati F, Tofanelli M, Tirelli G (2022). The immune microenvironment of HPV-positive and HPV-negative oropharyngeal squamous cell carcinoma: a multiparametric quantitative and spatial analysis unveils a rationale to target treatment-naive tumors with immune checkpoint inhibitors. J Exp Clin Cancer Res.

[34] Almangush A, Jouhi L, Haglund C, Hagstrom J, Makitie AA, Leivo I (2023). Tumor-stroma ratio is a promising prognostic classifier in oropharyngeal cancer. Hum Pathol.

[35] Santoro A, Pannone G, Ninivaggi R, Petruzzi M, Santarelli A, Russo GM, Lepore S, Pietrafesa M, Laurenzana I, Leonardi R (2015). Relationship between CK19 expression, deregulation of normal keratinocyte differentiation pattern and high risk-human papilloma virus infection in oral and oropharyngeal squamous cell carcinoma. Infect Agent Cancer.

[36] Regenbogen E, Mo M, Romeiser J, Shroyer ALW, Escobar-Hoyos LF, Burke S, Shroyer KR (2018). Elevated expression of keratin 17 in oropharyngeal squamous cell carcinoma is associated with decreased survival. Head Neck.

[37] Rahrotaban S, Mahdavi N, Abdollahi A, Yazdani F, Kaghazloo A, Derakhshan S (2019). Carcinoma-associated fibroblasts are a common finding in the Microenvironment of HPV-positive Oropharyngeal squamous cell carcinoma. Appl Immunohistochem Mol Morphol.

[38] Mohamed H, Haglund C, Jouhi L, Atula T, Hagstrom J, Makitie A (2020). Expression and role of E-Cadherin, beta-catenin, and vimentin in human papillomavirus-positive and human papillomavirus-negative Oropharyngeal squamous cell carcinoma. J Histochem Cytochem.

[39] Matlung SE, van Wilhelmina PM, Bovenschen N, van Baarle D, Willems SM (2016). Differences in T-cell infiltrates and survival between HPV + and HPV- oropharyngeal squamous cell carcinoma. Future Sci OA.

[40] De Meulenaere A, Vermassen T, Aspeslagh S, Deron P, Duprez F, Laukens D, Van Dorpe J, Ferdinande L, Rottey S (2017). Tumor PD-L1 status and CD8(+) tumor-infiltrating T cells: markers of improved prognosis in oropharyngeal cancer. Oncotarget.

[41] De Meulenaere A, Vermassen T, Aspeslagh S, Zwaenepoel K, Deron P, Duprez F, Rottey S, Ferdinande L (2017). Prognostic markers in oropharyngeal squamous cell carcinoma: focus on CD70 and tumour infiltrating lymphocytes. Pathology.

[42] Wuerdemann N, Gultekin SE, Putz K, Wittekindt C, Huebbers CU, Sharma SJ, Eckel H, Schubotz AB, Gattenlohner S, Buttner R et al. PD-L1 expression and a high Tumor infiltrate of CD8 + lymphocytes predict outcome in patients with Oropharyngeal squamous cells Carcinoma. Int J Mol Sci 2020, 21(15). 10.3390/ijms21155228 PMC7432501 32718057

[43] Kemnade JO, Elhalawani H, Castro P, Yu J, Lai S, Ittmann M, Mohamed ASR, Lai SY, Fuller CD, Sikora AG (2020). CD8 infiltration is associated with disease control and tobacco exposure in intermediate-risk oropharyngeal cancer. Sci Rep.

[44] Lechien JR, Descamps G, Seminerio I, Furgiuele S, Dequanter D, Mouawad F, Badoual C, Journe F, Saussez S. HPV involvement in the Tumor Microenvironment and Immune Treatment in Head and Neck squamous cell carcinomas. Cancers (Basel) 2020, 12(5). 10.3390/cancers12051060 PMC7281394 32344813

[45] Hatakeyama H, Mizumachi T, Sakashita T, Kano S, Homma A, Fukuda S (2014). Epithelial-mesenchymal transition in human papillomavirus-positive and -negative oropharyngeal squamous cell carcinoma. Oncol Rep.

[46] Lefevre M, Rousseau A, Rayon T, Dalstein V, Clavel C, Beby-Defaux A, Pretet JL, Soussan P, Polette M, Lacau Saint Guily J (2017). Epithelial to mesenchymal transition and HPV infection in squamous cell oropharyngeal carcinomas: the papillophar study. Br J Cancer.

[47] Toki MI, Cecchi F, Hembrough T, Syrigos KN, Rimm DL (2017). Proof of the quantitative potential of immunofluorescence by mass spectrometry. Lab Invest.

[48] Malay S, Chung KC (2012). The choice of controls for providing validity and evidence in clinical research. Plast Reconstr Surg.

[49] Nakanishi Y, Ochiai A, Kato H, Tachimori Y, Igaki H, Hirohashi S (2001). Clinicopathological significance of tumor nest configuration in patients with esophageal squamous cell carcinoma. Cancer.

[50] Crissman JD, Liu WY, Gluckman JL, Cummings G (1984). Prognostic value of histopathologic parameters in squamous cell carcinoma of the oropharynx. Cancer.

[51] Li X, Li C, Zhu G, Yuan W, Xiao ZA (2019). TGF-beta1 induces epithelial-mesenchymal transition of chronic sinusitis with nasal polyps through MicroRNA-21. Int Arch Allergy Immunol.

[52] Almangush A, Alabi RO, Troiano G, Coletta RD, Salo T, Pirinen M, Makitie AA, Leivo I (2021). Clinical significance of tumor-stroma ratio in head and neck cancer: a systematic review and meta-analysis. BMC Cancer.

[53] Karpathiou G, Vieville M, Gavid M, Camy F, Dumollard JM, Magne N, Froudarakis M, Prades JM, Peoc’h M (2019). Prognostic significance of tumor budding, tumor-stroma ratio, cell nests size, and stroma type in laryngeal and pharyngeal squamous cell carcinomas. Head Neck.

[54] Johnson DE, Burtness B, Leemans CR, Lui VWY, Bauman JE, Grandis JR (2020). Head and neck squamous cell carcinoma. Nat Reviews Disease Primers.

