# Supplementary material for: Analysis of cells of epithelial, connective tissue and immune differentiation in HPV-positive-, HPV-negative oropharyngeal carcinoma and normal oropharyngeal tissue by immunofluorescence multiplex image cytometry: a preliminary report
Source: BMC Cancer. 2023 Nov 27;23:1154. doi: 10.1186/s12885-023-11440-x (PMC10683252; doi:10.1186/s12885-023-11440-x)
Supplement: Supplementary file 1 — Supplementary Material 1 [file 12885_2023_11440_MOESM1_ESM.docx]

### Immunohistochemical details

Normal control tissue (figure 2a-l) displayed typical layered arrangement of a 100µm thick epithelial tissue with underlying connective tissue layer of the LP (figure 2b, 2h) penetrated by small blood vessels. A striated layer with tubular glands and secreting ducts dominates the most basal part in our samples. Mucosal epithelial cells express cytokeratin, and protein is expressed more abundantly in basal cells than apical cells. The very basal germinate cell layer contains least cytokeratin in its cytoplasm (figure 2g arrows). Epithelial cells of the glands as well as secreting ducts likewise express cytokeratin (figure 2a, 2c, 2g, 2i). Vimentin is expressed most in endothelial cells lining blood vessels as well as other cells of mesenchymal origin like fibroblast and smooth muscle cells (figure 2a, 2d, 2g, 2j). CD45/CD18 positive immune cells invade all connective tissue, but occasionally penetrate into epithelial layers (figure 2g, 2k). The striated order of tissue is dispersed into islands of tumor cell clusters with varying degree of immunoreactivity for cytokeratin (figure 2m-x). Within these tumor cell clusters more central epithelial cells often show higher intensity for cytokeratin staining than peripheral cells do, resembling the equal gradient of staining from basal to apical cells. Glands and secretory ducts were replaced by tumor cell clusters and connective tissue. Immunoreactivity for vimentin increased considerable. Increased blood vessels sprouting into the tumor nest may account most for this staining patter (figure 2m, 2p, 2s, 2v). Visually no difference in overall staining intensity was found for CD45/CD18 immune cells (figure 2m, 2q, 2s, 2w).

Some representative sections for patients with HPV- OPSCC (figure 1a-l) and patients with HPV+ OPSCC (figure 1m-x) in our study display major difference in the immunostaining pattern. HPV- tumor cell clusters frequently show only little immunoreactivity for cytokeratin (figure 1a, 1c, 1g, 1i). The sharp borders between connective tissue and big epithelial layers are increasingly replaced by smaller aggregations of tumor cells that leave space for more connective tissue in between (figure 1b, 1h). Vimentin as well as CD45/CD18 immunoreactivity in HPV+ does not differ visually from HPV- samples (Vimentin: figure 1a, 1d, 1g, 1j; CD45/CD18: figure 1a, 1e, 1g, 1k) and HPV- samples. HPV+ samples often present with higher immunoreactivity for cytokeratin in epithelial tumor cell clusters and display considerably less cytokeratin negative, “anaplastic” cells. Immunoreactivity levels range from basal layers of normal mucosal epithelia (figure 1e) as strong as the most differentiated apical layers in a mucosal layer of the same section. The borders between tumor cell clusters and stroma may not appear very sharp in H.E. stainings (figure 1n, 1t), but present as a marked front of cytokeratin positive tumor cells with well differentiated cytokeratin positive cells.
